# Editorial: The genetics and epigenetics of mental health

**DOI:** 10.3389/fgene.2024.1402495

**Published:** 2024-04-09

**Authors:** Gabriela Canalli Kretzschmar, Angelica Beate Winter Boldt, Adriano D. S. Targa

**Affiliations:** ^1^ Instituto de Pesquisa Pelé Pequeno Príncipe, Curitiba, Brazil; ^2^ Faculdades Pequeno Príncipe, Curitiba, Brazil; ^3^ Department of Genetics, Federal University of Parana, Post-graduation Program in Genetics, Curitiba, Brazil; ^4^ Translational Research in Respiratory Medicine, Hospital Universitari Arnau de Vilanova-Santa Maria, Biomedical Research Institute of Lleida (IRBLleida), Lleida, Spain; ^5^ CIBER of Respiratory Diseases (CIBERES), Institute of Health Carlos III, Madrid, Spain

**Keywords:** methylation, GWAS-genome-wide association study, microbiome & dysbiosis, poligenic risk score, neurological conditions, epigenome, genome

Mental health conditions cover a broad spectrum of disturbances, including neurological and substance use disorders, suicide risk, and associated psychosocial, cognitive, and intellectual disabilities (WHO, 2022). Despite a substantial amount of evidence, the interaction of genetic variants, epigenetic mechanisms, and environmental risk factors involved in mental health is poorly understood. Through distinct perspectives and different experimental approaches, the genetics and epigenetics of mental health were addressed in seven relevant articles included in this Research Topic, briefly summarized below.

Stress has severe consequences on the epigenome, but the timing of its occurrence, as well as the intensity and number of events, are critical for the severity of mental health symptoms. In particular, Serpeloni et al. demonstrated that stress generated in the form of intimate partner violence (IPV) during and/or after pregnancy impacts the offspring’s epigenome, shaping its resilience. They observed that individuals exposed to maternal IPV after birth presented psychiatric issues similar to their mothers, with different outcomes if the exposure to maternal IPV occurred both prenatally and postnatally. Prenatal IPV was associated with differential methylation in CpG sites in the genes encoding the glucocorticoid receptor (*NR3C1*) and its repressor FKBP51 (*FKBP5*), associated with the ability to terminate hormonal stress responses. Also considering early-life experiences and data from 2008 to 2016 of the Health and Retirement Study, Shin et al. concluded that early life experiences and relationships have a significant influence, attenuating or exacerbating the risk of suffering from mental health problems among individuals with a higher polygenic risk score predisposing to autism.

Environmental and developmental factors are also strongly linked to obsessive-compulsive disorder (OCD). They may explain the apparent discrepancy between the relatively high heritability scores and the inconsistent results found in genetic association studies, owing to their impact on gene expression and regulation. Based on this, Deng et al. stratified OCD patients by the age of disease onset. The findings revealed associations between the early onset and variants of genes whose products play a role in neural development, corroborating the age-associated genetic heterogeneity of OCD.

Further exploring environmental and genetic etiological clues, Li et al. used genome-wide association study (GWAS) data to calculate polygenic risk scores for salivary and tongue dorsum microbiomes associated with anxiety and depression. Additionally, causal relationships between the oral microbiome, anxiety, and depression were detected through Mendelian randomization, unraveling potential pathogenic mechanisms and interventional targets. Constructing a similar line of evidence, Becerra et al. found associations between the epigenetic regulation of inflammatory processes, the composition of gut microbiome, and modified Rosenberg self-esteem scores in samples from the Native Hawaiian and other Pacific Islander (NHPI) populations, which present a high prevalence and mortality from chronic and immunometabolic diseases, as well as mental health problems. This warrants further investigation into the relationship of microbiota to brain activity and mental health.

There is a lot of debate regarding suicidal behavior and its relationship with psychiatric disorders, but the extent to which they share the same genetic architecture is unknown. This Research Topic was investigated by Kootbodien et al. through the use of genomic structural equation modeling and Mendelian randomization with a large genomic dataset. The authors observed a strong genetic correlation between suicidal ideation, attempts, and self-harm, as well as a moderate to strong genetic correlation between suicidal behavioral traits and a range of psychiatric disorders, most notably major depressive disorder, involving pathways related to developmental biology, signal transduction, and RNA degradation. In conclusion, the study provided evidence of a shared etiology between suicidal behavior and psychiatric disorders, with overlapping pathophysiological pathways.

Malekpour et al., in their investigation of psychogenic non-epileptic seizures (PNES), also uncovered shared pathways with psychiatric conditions. PNES, the most prevalent non-epileptic disorder among patients referring to epilepsy centers, carries a mortality rate akin to drug-resistant epilepsy. Employing a systems biology approach, the authors pinpointed several key components influencing the disease pathogenesis network. These include brain-derived neurotrophic factor (BDNF), cortisol, norepinephrine, proopiomelanocortin (POMC), neuropeptide Y (NPY), the growth hormone receptor signaling pathway, phosphatidylinositol 3-kinase (PI3K)/protein kinase B (AKT) signaling, and the neurotrophin signaling pathway.

In general, these studies have some limitations: small sample sizes, leading to low statistical power in some cases, environmental confounding factors (such as diet and physical activity), which were not considered in the microbiome studies, incomplete phenotype descriptions, and partial coverages of human genetic diversity. Childhood adversities and adult comorbidities are among the variables that were not controlled for as possible causes of the investigated psychiatric and neurological disorders, and some results still claim for functional studies to be validated. Thus, the findings brought more elaborated questions, each of which shed some light on knowledge gaps that remain very difficult to fill. How do early-life epigenetic processes regulate our mental health resilience and disease resistance? What is the role of the microbiome in this process and how do genetic variants influence its composition? How does the impact of all these elements shape the resistance of human populations to psychiatric and neurological diseases and, most importantly, translate into public health measures in the future? We hope to engage more researchers in the pursuit of these answers.

